# Recombinant duck enteritis virus harboring the hemagglutinin genes of influenza virus rapidly induces specific cellular immunity in ducks

**DOI:** 10.1128/jvi.02014-25

**Published:** 2025-12-30

**Authors:** Yubo Zhao, Qi Ma, Chenchen Jiao, Jing Liu, Xiaoyu Zhang, Pucheng Chen, Zhiyuan Wen, Yongping Jiang, Xianying Zeng, Guohua Deng, Jianzhong Shi, Yanbing Li, Guobin Tian, Hualan Chen, Jinxiong Liu

**Affiliations:** 1 State Key Laboratory of Animal Disease Control and Prevention, Harbin Veterinary Research Institute, Chinese Academy of Agricultural Sciences 12661, Harbin, People's Republic of China; 2 National Poultry Laboratory Animal Resource Center, Harbin Veterinary Research Institute, Chinese Academy of Agricultural Sciences 12661, Harbin, People's Republic of China; Fred Hutchinson Cancer Center Vaccine and Infectious Disease Division, Seattle, Washington, USA

**Keywords:** duck enteritis virus, avian influenza, vaccine, innate immunity, cellular immunity

## Abstract

Given that ducks serve as critical reservoirs for avian influenza viruses, achieving high immune coverage in duck flocks is essential for preventing the transmission of avian influenza viruses from wild birds to domestic poultry. Duck enteritis is the most important infectious disease that must be prevented with a live-attenuated vaccine in duck breeding. Therefore, we previously constructed a recombinant duck enteritis virus (DEV), rDEV-dH5/H7, which carries the hemagglutinin (HA) genes of two H5 viruses and an H7 influenza virus. It could induce rapid and complete protection against both lethal DEV and H5 and H7 viruses as early as 7 days post-prime vaccination, although almost no antibodies were detected in ducks at this time. In the present study, we demonstrated that rDEV-dH5/H7 immediately initiated innate immune responses and T-cell proliferation in ducks. The expression levels of IFN-γ and granzyme A were markedly upregulated at 5–7 days post-prime vaccination. The percentages of CD3^+^ and CD3^+^CD8^+^ T cells in peripheral blood mononuclear cells significantly increased from 7 days post-prime vaccination. Moreover, HA-specific CD8^+^ and CD4^+^ T cells, as well as those induced by DEV virion, were significantly higher than those in control animals from 7 days post-prime vaccination. Together, the quick response of specific T cells and the innate immune response induced by rDEV-dH5/H7 may confer rapid immune protection against lethal influenza virus and DEV.

**IMPORTANCE:**

Previously, we found that recombinant duck enteritis virus, rDEV-dH5/H7, could induce rapid and robust dual protection against lethal DEV and highly pathogenic avian influenza viruses as early as day 7 post-prime vaccination, although almost no antibodies could be detected at this time. In the present study, we reveal that cell-mediated immunity plays a critical role through early upregulation of IFN-γ/granzyme A pathways and robust hemagglutinin (HA)-specific T-cell responses and drives protection even in the absence of detectable antibodies. This is the first mechanistic evidence showing DEV-vectored vaccines activate the robust proliferation of T lymphocytes and HA-specific T-cell responses. Our findings fundamentally advance the understanding of DEV-vectored vaccines, offering new insight for recombinant DEV vaccine design.

## INTRODUCTION

Duck virus enteritis, an acute and contagious disease that can be 100% lethal in ducks, is a worldwide disease caused by duck enteritis virus (DEV), a member of *Herpesviridae* and the subfamily *Alphaherpesvirinae* (1, 2). A live-attenuated DEV vaccine was successfully developed in the 1960s by attenuating the virulence of a lethal DEV strain through continuous passages in chicken embryos (3–5). Since then, attenuated DEV vaccines have been widely used to control duck viral enteritis, and these vaccines have provided adequate and satisfactory protective effects in ducks. In China, over 5 billion ducks are raised annually, accounting for more than 70% of the total duck breeding worldwide (6). Meanwhile, according to the data of the National Veterinary Drug Basic Information Database of China, 4.08–8.016 billion doses of the live-attenuated DEV vaccine were issued in 2024. This indicates that the vaccination coverage rate against DEV among China’s duck flocks is very considerable. In previous studies, we reported that the attenuated DEV vaccine used in China was a promising vaccine vector for developing polyvalent live vaccines against avian influenza viruses (7–9).

Ducks play a key role in the maintenance and transmission of avian influenza viruses in nature (10); therefore, the control of avian influenza viruses in ducks is critical for eradicating avian influenza from poultry. Because many influenza viruses that kill chickens are not lethal to ducks, farmers are reluctant to use inactivated avian influenza virus vaccines in ducks. Large numbers of unvaccinated ducks play an important role in the transmission of influenza viruses from wild birds to domestic poultry, posing a significant challenge for the effective control of avian influenza. To solve this problem, we developed a new vaccine strategy that protects against both the deadly pathogen in ducks, DEV, and the avian influenza virus circulating in domestic poultry to control infection and prevent the spread of avian influenza virus in ducks. Therefore, we constructed a recombinant DEV, rDEV-dH5/H7, an attenuated DEV vaccine for use as a vector. rDEV-dH5/H7 carries the hemagglutinin (HA) genes of two H5 viruses (GZ/S4184/17 [H5N6] [clade 2.3.4.4 h] and LN/SD007/17 [H5N1] [clade 2.3.2.1d]) and an H7 virus (GX/SD098/17 [H7N9]) (9). Animal studies revealed that rDEV-dH5/H7 induced strong and long-lasting hemagglutinin inhibition (HI) antibodies against H5 and H7 viruses post-booster and provided complete protection against challenges with lethal DEV and homologous and heterologous highly pathogenic H5 and H7 influenza viruses in ducks. Importantly, rDEV-dH5/H7 induced rapid, complete protection against both lethal DEV and influenza viruses as early as 7 days post-prime vaccination, although no neutralizing antibodies against DEV or almost no HI antibodies against H5 and H7 viruses were detected in ducks at this time (9). This phenomenon was also observed with the previously constructed rDEVus78HA (7, 8).

Both cellular and humoral immunity are involved in protection against lethal DEV (11–15), and live-attenuated DEV vaccines can induce quick protection (16, 17). The early immune responses and immunological mechanisms induced by attenuated DEV vaccines and their recombinant vaccines are poorly understood, especially in terms of cellular immune-related mechanisms. In this study, we comprehensively sketched the profiles of cytokine expression, the proliferation of CD3^+^, CD8^+^, and CD4^+^ T cells, and the specific T-cell responses induced by rDEV-dH5/H7 and the parental vector virus.

## MATERIALS AND METHODS

### Viruses and vaccines

The recombinant virus rDEV-dH5/H7 was constructed previously (9), and the attenuated DEV vaccine AV1222 strain (vDEV) was obtained from the China Veterinary Culture Collection. rDEV-dH5/H7 and vDEV were propagated in specific pathogen-free (SPF) chicken embryo fibroblasts. The avian influenza viruses GZ/S4184/17(H5N6), LN/SD007/17(H5N1), GX/SD098/17(H7N9), and WSN/1933 (H1N1) and the La Sota strain of Newcastle disease virus were propagated in the allantoic cavities of 10-day-old SPF embryonated chicken eggs. Peripheral blood mononuclear cells (PBMCs) stimulated with antigens for vDEV, influenza viruses, or Newcastle disease virus, which had been inactivated by β-propanolactone at a final concentration of 0.5% at room temperature for 48 h and purified by sucrose density gradient centrifugation, and then stored at −80°C.

The H1, H5, and H7 avian influenza virus antigens used in this study were cultured and inactivated in a biosafety level 3 (BSL-3) laboratory. The La Sota strain of Newcastle disease virus, vDEV, and rDEV-dH5/H7 is attenuated vaccine strains, and these pathogens were handled and operated in a BSL-2 laboratory.

### Quantitative PCR of cytokines

Three groups of SPF Shaoxing ducks (*Anas platyrhynchos*) were intramuscularly (i.m.) administered two doses (0.1 mL each) of 10^5^ TCID_50_ rDEV-dH5/H7, vDEV, or an equivalent volume of phosphate-buffered saline (PBS) at 4 and 7 weeks of age. Then, 4–5 ducks from each group were randomly selected and euthanized at designated time points from day 1 to day 28 post-prime vaccination. From each duck, 10^6^ PBMCs and 0.1 mg tissue of the bursa of Fabricius (BF), spleen, and lung were collected for quantification of ten cytokines. The mRNA levels of IFN-α, IFN-β, IFN-γ, granzyme A, IL-1β, IL-2, IL-4, IL-6, IL-8, and IL-15 were detected by real-time quantitative PCR (RT-qPCR). Recombinant pMD18-T vector plasmids carrying cytokine genes were used as references to calculate the absolute copy number. The primers used for cytokine RT-qPCR amplification and standard plasmid construction are listed in Table 1. Briefly, total cellular RNA was extracted using an RNA simple Total RNA Kit (TIANGEN Biotech, DP419), and the RNA was reverse transcribed into cDNA with a Prime Script RT reagent Kit (TaKaRa, RR037A) according to the manufacturer’s instructions. RT-qPCR was performed with TB Green Premix Ex Taq (TaKaRa, RR820A) using an Applied Biosystems QuantStudio 5 Real-Time PCR System (Thermo Fisher Scientific). The RT-qPCR program was as follows: initial denaturation at 95°C for 30 s, followed by 40 cycles of denaturation at 95°C for 5 s and annealing/extension at 60°C for 34 s, with end-point melt curve analysis. Cytokine mRNA expression levels were quantified by absolute quantitative PCR. Fold changes in the two vaccination groups relative to the control group were then calculated, log10-transformed, and visualized as a heatmap.

**TABLE 1:** Oligonucleotide sequences used for the detection of cytokine mRNA by RT-PCR

Target	Accession number	Oligonucleotide sequences used for RT-PCR（5′−3′）	Oligonucleotide sequences used for plasmid construction（5′−3′）
IFN-α	EF053034	RT-F: TCCTGGACACCAACGAC	pMD18T-F: CGCCAACGCCTTCTCCTG
		RT-R: TGGATGCAGCCGAAGTA	pMD18T-R: CGGTGGATGCGCTGGAA
IFN-β	KM032183	RT-F: ACCACTACATCTACCACCTCG	pMD18T-F: CCACCACCACCAGCCATCT
		RT-R: CTTGCTCGGCATCCACT	pMD18T-R: CCTCTTGCTCGGCATCCACT
IFN-γ	AJ012254	RT-F: AACGCAAGGCTGTGAGTGAG	pMD18T-F: AACGCAAGGCTGTGAGTGAG
		RT-R: ACTGGCTCCTTTTCCTTTTGG	pMD18T-R: ACTGGCTCCTTTTCCTTTTGG
Granzyme A	XM027446934.2	RT-F: GCTGCTGTCATCCTC	pMD18T-F: ATGGGAGCTTTCTTTACCTTG
		RT-R: CCATCCTGCTACTCTAC	pMD18T-R: TCAAAAGCCGGTCTGTAAGTCT
IL-1β	DQ393268	RT-F: TGGGCATCAAGGGCTACAAG	pMD18T-F: ATCCAGCCAGAAAGTGAGG
		RT-R: GCTGTCGATGTCCCGCATGA	pMD18T-R: AGGCGGTAGAAGATGAAGC
IL-2	AF294323	RT-F: GCCAAGAGCTGACCAACTTC	pMD18T-F: GAGCACCTCTATCAGA
		RT-R: ATCGCCCACACTAAGAGCAT	pMD18T-R: AAGTTGTTGGCATT
IL-4	KY427739.1	RT-F: CCTTCTCCGTCCTGCTC	pMD18T-F: CCTTCTCCGTCCTGCTC
		RT-R: CTCGTTGGAGGGTTCTGT	pMD18T-R: CTCGTTGGAGGGTTCTGT
IL-6	AB191038	RT-F: AGATGGTGATAAATCCTGATGA	pMD18T-F: GGAGCCGAAGAAGAAGAGCC
		RT-R: CGGTTTTCTCCATAAATGAAGT	pMD18T-R: GCCCGAATTAAAACATTCAGACA
IL-8	NM001310420.1	RT-F: AAGTTCATCCACCCTAAATC	pMD18T-F: CTGGCTCTTCTCTTGATTTCC
		RT-R: GCATCAGAATTGAGCTGAGC	pMD18T-R: GGGGTCCAAGCACACCT
IL-10	JN786941.1	RT-F: AGCAGCGAGCACCACCA	pMD18T-F: ATGAGAACCTGCTGCGTGG
		RT-R: TGCCGTTCTCGTTCATCTTT	pMD18T-R: TCACTTCCTCCTCTTCATCAG
IL-15	XM005030958.4	RT-F: ACGATGTAGTTGCCTGGTT	pMD18T-F: ACGATGTAGTTGCCTGGTT
		RT-R: GGATGTCTTGATCTGCTCC	pMD18T-R: GGATGTCTTGATCTGCTCC

### T-cell expansion induced by rDEV-dH5/H7

Three groups of SPF Shaoxing ducks were i.m. administered two doses (0.1 mL each) of 10^5^ TCID_50_ rDEV-dH5/H7, vDEV, or an equivalent volume of PBS at 4 and 7 weeks of age. Then, PBMCs were isolated from ducks in each group at different time points from day 0 (before inoculation) to day 91 post-prime vaccination. The PBMCs were plated in 1.5 mL centrifuge tubes (10^6^ cells/tube) and fixed with 100 μL of 0.3% paraformaldehyde for 20 min at room temperature and subsequently stained for 30 min at room temperature with either mouse anti-duck CD4 mAb (Du CD4-2; Bio-Rad, MCA2478) or mouse anti-duck CD8 mAb (Du CD8-1; Bio-Rad, MCA2479) diluted in PBS containing 0.05% Tween-20. After being washed twice, labeled PBMCs were further incubated with Alexa Fluor 647-conjugated goat anti-mouse IgG (H + L) (Thermo Fisher Scientific, A-21235) in the dark at room temperature for 30 min. Then, they were stained with an FITC-conjugated rat anti-CD3 mAb (CD3-12; Abcam, ab11089) for 30 min at room temperature. A minimum of 10,000 cells was acquired for flow cytometry analysis. Flow cytometry was performed with a Cytomics FC500 (Beckman), and the data were analyzed using FlowJo v10 software (Tree Star).

The rat anti-CD3 mAb (CD3-12; Abcam, ab11089) was confirmed that it can react with duck CD3 by western blotting and flow cytometry. Briefly, the genes of duck CD3ε (GeneID: 101792798, NM 001310778.1) and mouse CD3ε (Gene ID: 12501, NM 007648.5) were amplified with a C-terminal Flag-tag and cloned into pGAGGS vector (Solarbio, VT000109). Subsequently, HEK-293T cells were transfected with the resulting plasmids, respectively. The expression of duck and mouse CD3ε in the cells was identified by western blotting, and the cross-reactivity and specificity of the CD3 mAb binding to HEK-293T cells expressing either duck or mouse CD3ε were further confirmed by flow cytometry. The CD3 mAb was also further validated by flow cytometry using PBMCs from ducks and chickens, as well as splenocytes from mice, confirming its reactivity with lymphocytes from all three species.

### Specific T-cell responses

Three groups of SPF Shaoxing ducks were i.m. administered two doses (0.1 mL each) of 10^5^ TCID_50_ rDEV-dH5/H7, vDEV, or an equivalent volume of PBS at 4 and 7 weeks of age. PBMCs were isolated from ducks in each group at different time points from day 0 (before inoculation) to day 31 post-prime vaccination. The PBMCs were cultured for 16 h in 96-well plates at a concentration of 2 × 10^6^ cells/well in 200 μL of RPMI 1640 medium supplemented with 10% FBS, 2 mM L-glutamine, 1 M HEPES, 100 mM sodium pyruvate, and 1 μM GolgiStop (BD Pharmingen). The purified inactivated virus antigens, vDEV, GZ/S4184/17(H5N6), LN/SD007/17(H5N1), GX/SD098/17(H7N9), and a mixture of the three influenza viruses of equal quality were added to the cells described above for five stimulated groups at a final concentration of 100 μg/mL. Moreover, inactivated H1N1 virus (WSN/1933) and Newcastle disease virus (La Sota strain) were used as the uncorrelated control group at a final concentration of 100 μg/mL, RPMI 1640 medium was used as the negative control group, and a mixture of phorbol 12-myristate 13-acetate (PMA, 50 ng/mL) and ionomycin (IONO, 1 μM) was used as the positive control. After 12 h of culture, the PBMCs were collected and incubated with Cytofix/Cytoperm (BD Pharmingen) for 20 min at 4°C and then stained with a rabbit anti-duck IFN-γ mAb (prepared in our laboratory) at room temperature for 30 min. After washing with Perm/Wash buffer (BD-Pharmingen) twice, labeled cells were further incubated with Alexa Fluor 647-conjugated goat anti-rabbit IgG (H + L) (Thermo Fisher Scientific, A-21235) in the dark at room temperature for 30 min. Then, the labeled cells were stained with 100 μL of antibody cocktail at room temperature for 30 min. The antibody cocktail included an FITC-conjugated anti-CD3 mAb (CD3-12; Abcam, ab11089) together with a PE-conjugated anti-duck CD4 mAb (Du CD4-2; Bio-Rad, MCA2478) or a PE-conjugated anti-duck CD8 mAb (Du CD8-1; Bio-Rad, MCA2479), which were labeled with a PE/R-phycoerythrin Conjugation Kit (Abcam, ab102918), respectively. A minimum of 50,000 cells was acquired for flow cytometry analysis. Flow cytometry was performed with an A60-Universal flow cytometer (Apogee), and the data were analyzed using FlowJo v10 software (Tree Star).

### Statistical analyses

Statistical analyses were performed using GraphPad Prism 10 software. Statistical significance was calculated using the Student’s two-tailed unpaired *t* test. The results were considered statistically significant if *P* < 0.05 (∗), *P*< 0.01 (∗∗), or *P*<0.001 (∗∗∗).

## RESULTS

### Cytokine responses induced by rDEV-dH5/H7

Innate immunity plays a critical role in inducing adaptive immunity and controlling the initial events of viral infection. Several pathogen recognition receptors are involved in recognition during lethal DEV infection in ducks, and they may cooperatively induce the expression of cytokines (18). To address the profile of cytokine reactions induced by rDEV-dH5/H7 and vDEV, the mRNA expression levels of 10 cytokines were quantified from 4 different tissues, namely, PBMCs, BF, spleen, and lung, of inoculated SPF ducks.

**Fig 1:**
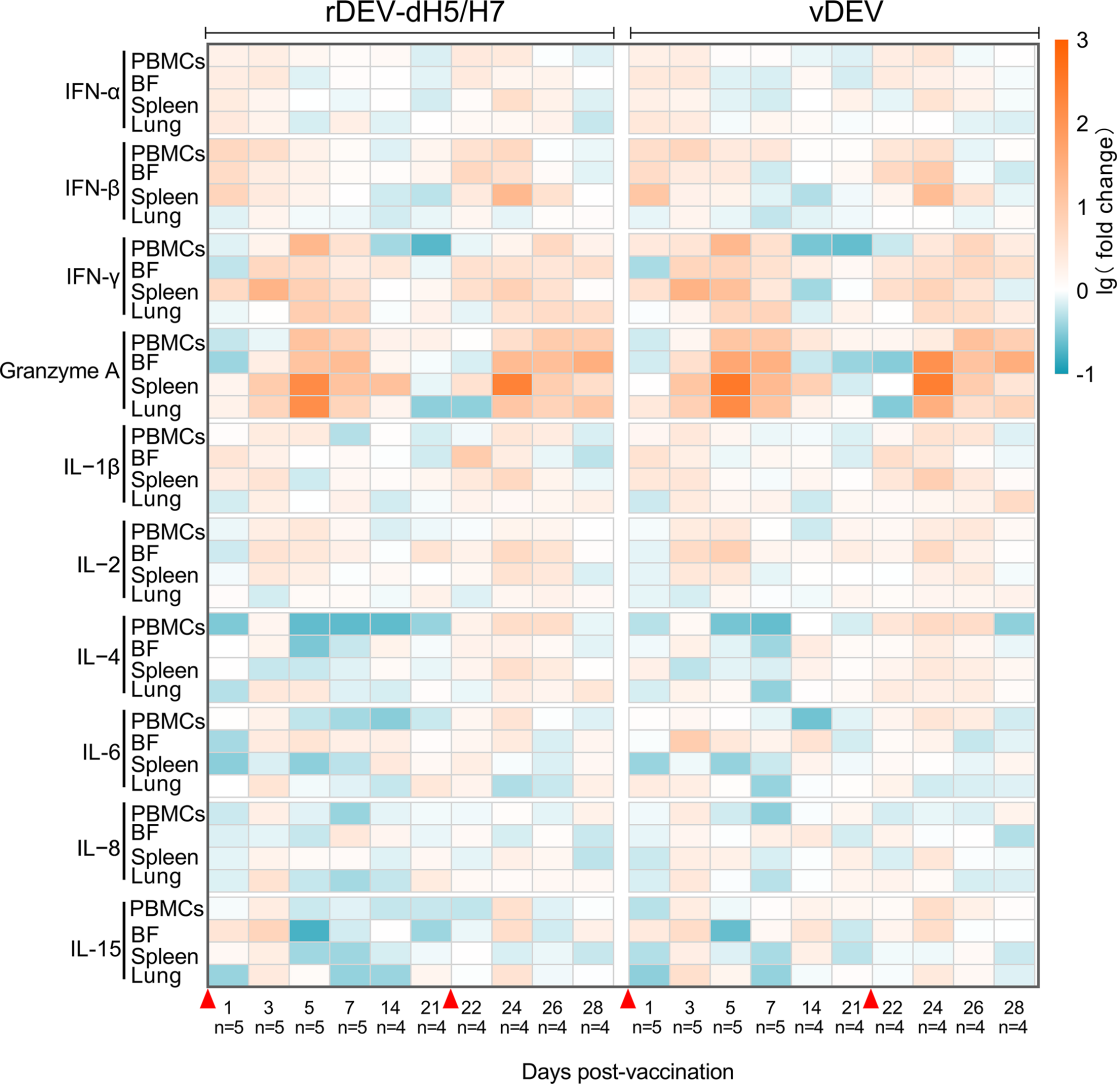
Expression of cytokines induced by rDEV-dH5/H7 and vDEV. Four-week-old ducks were inoculated i.m. with two doses of 10^5^ TCID_50_ of rDEV-dH5/H7 or vDEV at a 3-week interval. Heatmaps depicting the upregulated or downregulated mRNAs of differential cytokine expression between rDEV-dH5/H7-inoculated ducks and control ducks, and between vDEV-inoculated ducks and control ducks. The data were normalized using the log10-fold change. In the heatmaps, red denotes upregulated transcripts, blue denotes downregulated transcripts, and white denotes no expression change at the indicated times. The red triangles indicate the time points of inoculation.

Innate immune responses were immediately initiated after both rDEV-dH5/H7 and vDEV inoculation. The expression levels of the tested cytokine genes were mostly upregulated in the four tissues, and the cytokine expression pattern induced by rDEV-dH5/H7 was highly similar to that induced by vDEV (Fig. 1; Fig. S1). IFN-α and IFN-β were the earliest-acting cytokines, and their mRNAs were significantly upregulated in PBMCs and the BF at 1 and 3 days post-prime vaccination and were significantly upregulated at 1 day post-prime vaccination in the spleen. The IFN-α and IFN-β mRNAs returned to normal concentrations at 7 days post both prime and booster vaccination. Most obviously, IFN-γ and granzyme A were significantly upregulated in all four tissues at the early phase and mostly peaked at 5 days post-prime vaccination (Fig. 1; Fig. S1), and this might be related to the effect of the prime-boost immune response; the mRNA levels of both granzyme A and IFN-γ increased dramatically in most tissues on day 3 after boosting, reaching levels comparable to those at 5 days post-prime vaccination. The IFN-γ mRNA levels in the four tissues of both groups of vaccine-inoculated ducks were 4.23- to 21.70-fold higher than those in the control ducks at 5 days post-prime vaccination. The granzyme A mRNA levels in the four tissues of both groups of vaccine-inoculated ducks were 12.98- to 149.07-fold higher than those in the control ducks at 5 days post-prime vaccination, and the mRNA levels in the three tissues of BF, spleen, and lung were 11.41- to 253.19-fold higher than those in the control ducks on day 3 after boosting. The mRNA levels of IFN-γ and granzyme A then returned to normal concentrations in the tested tissues at 14 days post-prime vaccination. Additionally, the mRNA levels of IL-1β and IL-2 were upregulated in some of the tissues at 3–5 days post both prime and booster vaccination, while the mRNA levels of IL-4, IL-6, IL-8, and IL-15 showed diverse trends across different tissues and time after vaccination. Specifically, the IL-4 mRNA levels in the PBMCs of the rDEV-dH5/H7-inoculated ducks were 4.55- to 4.68-fold lower than those in the control ducks between 5 and 14 days post-prime vaccination (Fig. 1; Fig. S1).

### T-cell expansion induced by rDEV-dH5/H7

**Fig 2:**
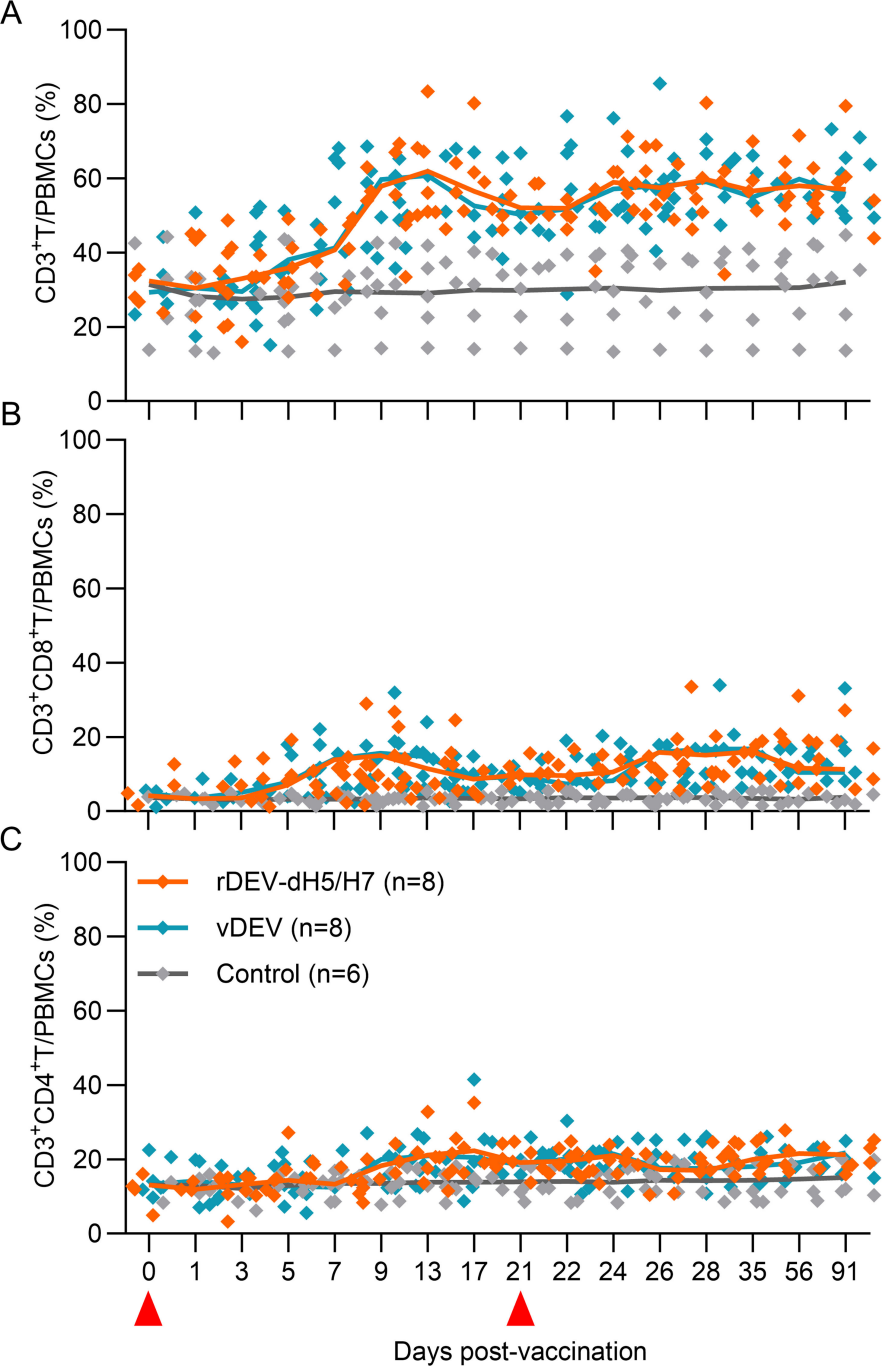
Expansion of T cells induced by rDEV-dH5/H7 and vDEV. Four-week-old ducks were inoculated i.m. with two doses of 10^5^ TCID_50_ of rDEV-dH5/H7 or vDEV at a 3-week interval. The percentages of (**A**) CD3^+^ T cells, (**B**) CD3^+^CD8^+^ T cells, and (**C**) CD3^+^CD4^+^ T cells among the PBMCs were analyzed by flow cytometry. Data are presented as means and plotted with connecting lines. Each data point represents one sample value. The red triangles represent the time points of inoculation.

To evaluate the T-cell response induced by rDEV-dH5/H7 and vDEV, the rat anti-CD3 mAb was first validated that it can react with duck CD3 (Fig. S2). Subsequently, we determined the percentages of CD3^+^, CD3^+^CD8^+^, and CD3^+^CD4^+^ T-cell subset populations in the PBMCs of inoculated ducks. A representative gating strategy for T-cell analysis is shown in Fig. S3. As shown in Fig. S3, CD3^+^, CD3^+^CD8^+^, and CD3^+^CD4^+^ T cells proliferated rapidly in the rDEV-dH5/H7- and vDEV-inoculated ducks. The percentage of CD3^+^/PBMCs increased from day 3 to day 13 post-prime vaccination, with the mean percentages increasing dramatically from 33.0% and 33.4% at 3 days post-prime vaccination to 62.0% and 59.6% at 13 days post-prime vaccination in rDEV-dH5/H7- and vDEV-inoculated ducks, respectively. The percentages were maintained at high levels in both groups of vaccine-inoculated ducks until the end of the trial at 91 days post-prime vaccination, with mean values consistently exceeding 57.1%. In contrast, the percentage of CD3^+^/PBMCs in the control animals was maintained between 27.5% and 32.0% (Fig. 2A). The percentages of CD3^+^CD8^+^/PBMCs in the rDEV-dH5/H7- and vDEV-inoculated ducks also increased rapidly from day 3 to day 9 post-prime vaccination, with the mean percentages increasing from 4.0% and 4.0% at 3 days post-prime vaccination to 16.2% and 16.2% at 9 days post-prime vaccination, respectively. After boosting, the percentages of CD3^+^CD8^+^/PBMCs increased to 18.0% and 18.1% at 7 days post-booster vaccination. Thereafter, the ratio decreased slightly but remained at a high level until the end of the trial, and the mean percentages were still higher than 13.8% in both groups of vaccine-inoculated ducks. The percentage of CD3^+^CD8^+^ PBMCs in the control animals was maintained between 3.1% and 4.0% (Fig. 2B). The percentages of CD3^+^CD4^+^/PBMCs in the rDEV-dH5/H7- and vDEV-inoculated ducks continuously increased from day 7 to day 17 post-prime vaccination, with the mean percentages increasing from 13.3% and 13.4% at 7 days post-prime vaccination to 22.3% and 23.3% at 17 days post-prime vaccination, respectively. After boosting, the ratio increased slightly in both groups of vaccine-inoculated ducks and remained elevated throughout the trial period, with mean values consistently exceeding 20.7%. In contrast, the percentage of CD3^+^CD4^+^ PBMCs in the control animals was maintained between 12.2% and 15.1% (Fig. 2C).

These data showed that both rDEV-dH5/H7 and vDEV induced rapid T-cell proliferation, with no significant difference in the percentages of CD3^+^, CD3^+^CD8^+^, or CD3^+^CD4^+^ T cells between the two inoculated groups at all time points. The percentages of CD3^+^, CD3^+^CD8^+^, and CD3^+^CD4^+^ T cells in the PBMCs of both inoculated groups of ducks were significantly higher than those of the control ducks from 7, 7, and 9 days post-prime vaccination to the end of the trial (*P* < 0.05), respectively. At 7 days post-prime vaccination of rDEV-dH5/H7, the percentages of CD3^+^ and CD3^+^CD8^+^ T cells among the PBMCs increased to 40.8% and 12.9%, respectively, which were approximately 1.4- and 4.1-fold higher than those of the control animals. Moreover, the percentages of CD3^+^, CD3^+^CD8^+^, and CD3^+^CD4^+^ T cells in the PBMCs were approximately 2.1-, 4.2-, and 1.5-fold higher than those in the control animals at 13 days post-prime vaccination. These results indicated that cellular immunity may play a key role in inducing rapid protection against DEV and influenza challenges. Therefore, we further tested specific T-cell responses induced by rDEV-dH5/H7.

### Specific T-cell responses induced by rDEV-dH5/H7

To address the profiles of DEV- and HA-specific CD8^+^ and CD4^+^ T-cell responses induced by rDEV-dH5/H7 in ducks, we stimulated duck PBMCs with whole-virus antigens, including inactivated vDEV, GZ/S4184(H5N6), LN/SD007(H5N1), GX/SD098(H7N9), and a mixture of three influenza virus antigens, and intracellular staining for IFN-γ was performed. DEV- and HA-specific CD3^+^CD8^+^ and CD3^+^CD4^+^ T cells expressing IFN-γ^+^ were then quantified from stimulated PBMCs (Fig. S4).

**Fig 3:**
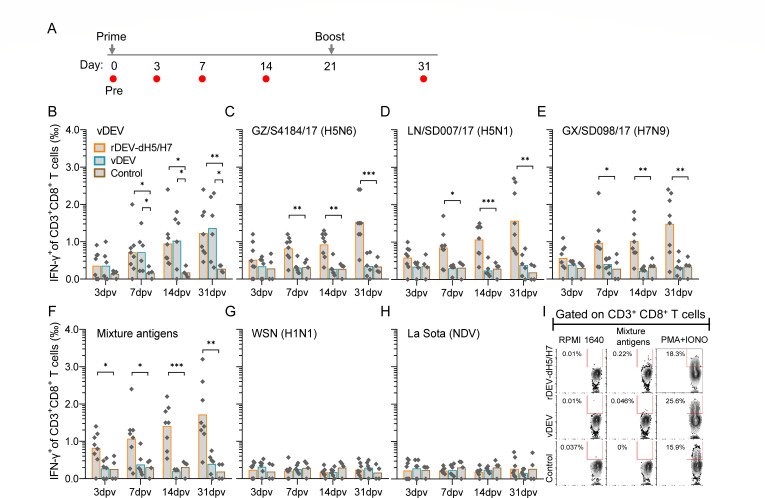
Specific CD8^+^ T-cell responses induced by rDEV-dH5/H7 and vDEV. (A) Timeline of inoculation and sample testing. Arrows indicate inoculation, and red circles indicate bleeds, tests, and post-vaccination time points. (**B**) Frequency of specific CD3^+^CD8^+^ T cells expressing IFN-γ^+^ induced by DEV virion. (**C–F**) Frequency of HA-specific CD3^+^CD8^+^ T cells expressing IFN-γ^+^. PBMCs were stimulated with single inactivated virus antigens: GZ/S4184 (H5N6), LN/SD007 (H5N1), GX/SD098 (H7N9), or a mixture of antigens of these three influenza virus antigens; CD3^+^CD8^+^IFN-γ^+^ T cells were then quantified. (**G and H**) Uncorrelated control. CD3^+^CD8^+^IFN-γ^+^ T cells were quantified after PBMCs were stimulated with WSN (H1N1) or La Sota (NDV). Data are presented as means in histograms. Each data point represents one sample value. Statistical significance: *P < 0.05, **P <0.01, and ***P < 0.001. *P* values were determined using a two-tailed unpaired Student’s *t* test. (**I**) Representative gating strategy for HA-specific CD3^+^CD8^+^ T cells expressing IFN-γ^+^ detected at 31 days post-prime vaccination, following stimulation with the influenza virus antigens mixture. RPMI 1640 and PMA plus ionomycin served as negative and positive controls, respectively.

**Fig 4:**
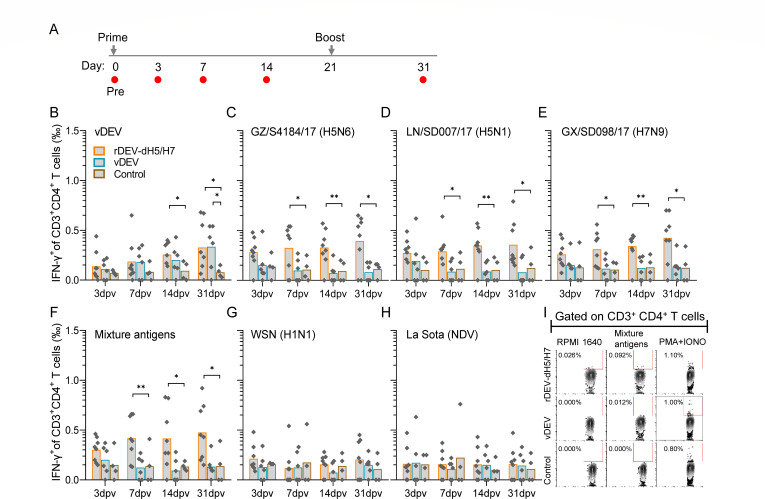
Specific CD4^+^ T-cell responses induced by rDEV-dH5/H7 and vDEV. (**A**) Timeline of inoculation and sample testing. Arrows indicate inoculation, and red circles indicate bleeds, tests, and post-vaccination time points. (**B**) Frequency of specific CD3^+^CD4^+^ T cells expressing IFN-γ^+^ induced by DEV virion. (**C–F**) Frequency of HA-specific CD3^+^CD4^+^ T cells expressing IFN-γ^+^. PBMCs were stimulated with single inactivated virus antigens: GZ/S4184 (H5N6), LN/SD007 (H5N1), GX/SD098 (H7N9), or a mixture of antigens of these three influenza virus antigens; CD3^+^CD4^+^IFN-γ^+^ T cells were then quantified. (**G and H**) Uncorrelated control. CD3^+^CD4^+^IFN-γ^+^ T cells were quantified after PBMCs were stimulated with WSN (H1N1) or La Sota (NDV). Data are presented as means in histograms. Each data point represents one sample value. Statistical significance: *P < 0.05, **P <0.01, and ***P < 0.001. *P* values were determined using a two-tailed unpaired Student’s *t* test. (**I**) Representative gating strategy for HA-specific CD3^+^CD4^+^ T cells expressing IFN-γ^+^ detected at 31 days post-prime vaccination, following stimulation with the influenza virus antigens mixture, RPMI 1640, and PMA plus ionomycin served as negative and positive controls, respectively.

Following stimulation with vDEV antigen, the IFN-γ^+^ cells within CD3^+^CD8^+^ T cells were increased immediately from 3 days post-prime vaccination in the rDEV-dH5/H7- and vDEV-inoculated ducks, becoming significantly higher than that in the control group animals at 7 days post-prime vaccination; the mean permillages were both 0.72‰ at 7 days post-prime vaccination (Fig. 3B). In parallel, the IFN-γ^+^ cell ratios among CD3^+^CD4^+^ T cells increased from 7 days post-prime vaccination in the rDEV-dH5/H7- and vDEV-inoculated ducks, showing a significant difference from controls at 14 days post-prime vaccination (Fig. 4B). After boosting, the IFN-γ^+^ cell ratios among CD3^+^CD8^+^ T cells and CD3^+^CD4^+^ T cells further increased. These findings demonstrated that both vaccines induced robust DEV-specific T-cell immunity, with no significant differences observed between rDEV-dH5/H7 and vDEV.

When PBMCs were stimulated with three single influenza virus antigens or their mixture, the IFN-γ^+^ cell within CD3^+^CD8^+^ and CD3^+^CD4^+^ T cells increased immediately in the rDEV-dH5/H7-inoculated ducks from 3 days post-prime vaccination (Fig. 3C through F and 4C through F). Notably, the IFN-γ^+^ cell permillages in CD3^+^CD8^+^ T cells were significantly higher than those of control animals in the three influenza virus mixture antigen-stimulated groups at 3 days post-prime vaccination (Fig. 3F), and the IFN-γ^+^ cell ratios were significantly higher than those in the control animals from 7 days post-prime vaccination in all four different antigen-stimulated groups (Fig. 3C through F). The IFN-γ^+^ cell permillages in CD3^+^CD8^+^ T cells gradually increased from 0.82‰ and 1.07‰ at 7 days post-prime vaccination to 1.48‰ and 1.72‰ at 10 days post-booster vaccination. The IFN-γ^+^ cell ratios in influenza virus mixture antigen-stimulated PBMCs were slightly higher than those in single virus antigen-stimulated PBMCs. Moreover, significantly greater numbers of IFN-γ^+^ cells among CD3^+^CD4^+^ T cells were also induced in the rDEV-dH5/H7-inoculated ducks than in the control ducks from 7 days post-prime vaccination, with 0.29‰–0.42‰ of total CD3^+^CD4^+^ T cells responding to four different antigens stimulated (Fig. 4C through F). The proportion of IFN-γ^+^CD3^+^CD4^+^ T cells was then maintained at a comparable level until 10 days post-booster vaccination.

Notably, no increase in the IFN-γ^+^ cell proportion among CD3^+^CD8^+^ or CD3^+^CD4^+^ T cells was observed in vDEV-inoculated ducks upon stimulation with any influenza virus antigens at all tested time points (Fig. 3C through F and 4C through F). Similarly, no response was observed following stimulation with H1N1 virus and NDV antigens (Fig. 3G and H, 4G and H). These data indicated that rDEV-dH5/H7 could induce obvious and rapid HA-specific T-cell immunity.

## DISCUSSION

In the present study, we found that innate immune responses and T-cell proliferation were immediately initiated after both rDEV-dH5/H7 and vDEV inoculation, and there was no significant difference between the animals inoculated with the two different vaccines. The expression levels of the 10 tested cytokine genes were mostly upregulated in the PBMCs, BF, spleen, and lung post-inoculation, with IFN-γ and granzyme A most obviously upregulated in all four tissues in the early phase of both-dose inoculations. At the same time, rDEV-dH5/H7 rapidly activated the CD3^+^ and CD3^+^CD8^+^ T-cell proliferative response starting at 3 days post-prime vaccination and activated the CD3^+^CD4^+^ T-cell proliferative response starting at 7 days post-prime vaccination. Its potency was comparable to vDEV, with no significant difference in the expansion of CD3^+^, CD3^+^CD8^+^, or CD3^+^CD4^+^ T cells in the PBMCs at all tested time points. Moreover, specific T-cell responses to DEV virion were activated immediately after inoculation with rDEV-dH5/H7 and vDEV, and specific CD8^+^ T cells were significantly higher than those in the control group from 7 days post-prime vaccination, with no difference between the two vaccines. HA-specific CD8^+^ and CD4^+^ T cells stimulated with three single influenza virus antigens or their mixture were also activated from 3 days post-prime vaccination after ducks were inoculated with rDEV-dH5/H7. Significant increases in HA-specific CD8^+^ and CD4^+^ T cells were observed at 7 days post-prime vaccination. Notably, HA-specific CD8^+^ T cells were significantly higher than those of control animals at 3 days post-prime vaccination in the three influenza virus mixture antigen-stimulated groups. After boosting, stronger HA-specific CD8^+^ and CD4^+^ T responses were observed. This study is the first time to clarify the innate immune responses and special T-cell proliferation in the early stage after vDEV and recombinant virus inoculation.

Since the 1960s, live-attenuated DEV vaccines have been demonstrated to elicit rapid protection and could be used for emergency immunization. Even after the diagnosis of lethal DEV infection in duck flocks, emergency vaccination with attenuated DEV could provide up to 95% protection (3, 16). Research has shown that the protection efficacy of vaccines reaches 60% (3/5) at 1 day post-prime vaccination (3), and complete protection can be obtained by 3 days post-prime vaccination (7). This rapid protection phenomenon is also observed with Marek’s disease vaccine (19). The exact mechanism of the rapid protection before 3 days post-prime vaccination is unknown, and interference may play a role in protection against lethal DEV (17). However, the quick protection against H5 and H7 virus challenge at 7 days post rDEV-dH5/H7 vaccination (9) is the result of adaptive immunity or HA-activated innate immunity. In this study, we found that inoculation with rDEV-dH5/H7 and vDEV triggered the release of IFN-γ and granzyme A at 5–7 days post-prime vaccination and also induced rapid T-cell proliferation. At 7 days post-prime vaccination, the percentages of CD3^+^ and CD3^+^CD8^+^ T cells in PBMCs were significantly higher than those in the control ducks; moreover, the percentages of specific CD8^+^ T cells to DEV virion and HA and the percentages of HA-specific CD4^+^ T cells in the inoculated animals were significantly higher than those in the control animals. T-cell-mediated immunity plays a dominant role in host defense against herpesviruses (20), and CD8^+^ T cells play a key role in clearing influenza virus infection and subsequent host recovery (21, 22). Although CD4^+^ T cells also have both cytotoxic and antiviral responses, they mainly provide helper effects to CD8^+^ T cells and B cells (23–26). IFN-*γ* is a major cytokine induced by helper T (Th1) and CD8^+^ T cells and plays a critical role in antiviral immunity. Granzyme A is expressed in CD8^+^ T and NK cells and primarily mediates perforin-dependent cytotoxic effects (27–29). Therefore, we speculate that the rapid response of specific T cells and the innate immune response induced by rDEV-dH5/H7 together confer rapid immune protection against lethal influenza virus and DEV.

DEV replicates primarily in the mucosal epithelial cells of the digestive tract and then spreads to the BF, thymus, spleen, and liver (26, 30). The epithelial cells, lymphocytes, and macrophages in these organs are the main target cells of viral replication (31, 32), and most of them are professional antigen-presenting cells (APCs). Therefore, when rDEV-dH5/H7 infects and undergoes replication cycles within host cells, it allows *de novo* synthesis of HA proteins in their native conformation. These HA antigens in the cytosol of APCs are subsequently presented to CD8^+^ T cells, and HA fragments of processed antigens on their surfaces activate CD4^+^ T cells. CD4^+^ T cells offer costimulatory signals for the priming of B cells and CD8^+^ T cells. rDEV-dH5/H7 could induce (cross-) protective immunity against heterologous H5 viruses (9), which may be correlated with HA-specific CD8^+^ T-cell responses because the HA stem contains conserved CD8^+^ T-cell epitopes (33, 34). Moreover, it may also be correlated with the presence of multiple conserved protective B-cell epitopes on HA proteins (35). Influenza viruses easily undergo antigenic variation during their circulation in nature. DEV shares similar target organs with most avian influenza viruses, and both replicate primarily in the intestinal tract of ducks (30, 36); therefore, the attenuated DEV vaccine is an ideal antigen delivery system for developing influenza virus vector vaccines.

## Data Availability

All data generated or analyzed during this study are included in this published article and its supplementary material.

## SUPPLEMENTAL MATERIAL

Fig. S1A-E jvi.02014-25-s0001.tif Cytokine expression induced by rDEV-dH5/H7 and vDEV.

Fig. S1F-G jvi.02014-25-s0002.tif Cytokine expression induced by rDEV-dH5/H7 and vDEV.

Fig. S2 jvi.02014-25-s0003.tif Cross-reactivity and specificity of rat anti-CD3 mAb CD3-12 with duck CD3.

Fig. S3 jvi.02014-25-s0004.tif Representative gating strategy for T-cell analysis.

Fig. S4 jvi.02014-25-s0005.tif Representative gating strategy for specific T-cell analysis.

Supplemental legends jvi.02014-25-s0006.docx Legends for Fig. S1 to S4.

ASM does not own the copyrights to Supplemental Material that may be linked to, or accessed through, an article. The authors have granted ASM a non-exclusive, world-wide license to publish the Supplemental Material files. Please contact the corresponding author directly for reuse.
