# Supplementary figures and images for: Recombinant duck enteritis virus harboring the hemagglutinin genes of influenza virus rapidly induces specific cellular immunity in ducks
Source: J Virol. 2025 Dec 30;100(2):e02014-25. doi: 10.1128/jvi.02014-25 (PMC12911906; doi:10.1128/jvi.02014-25)

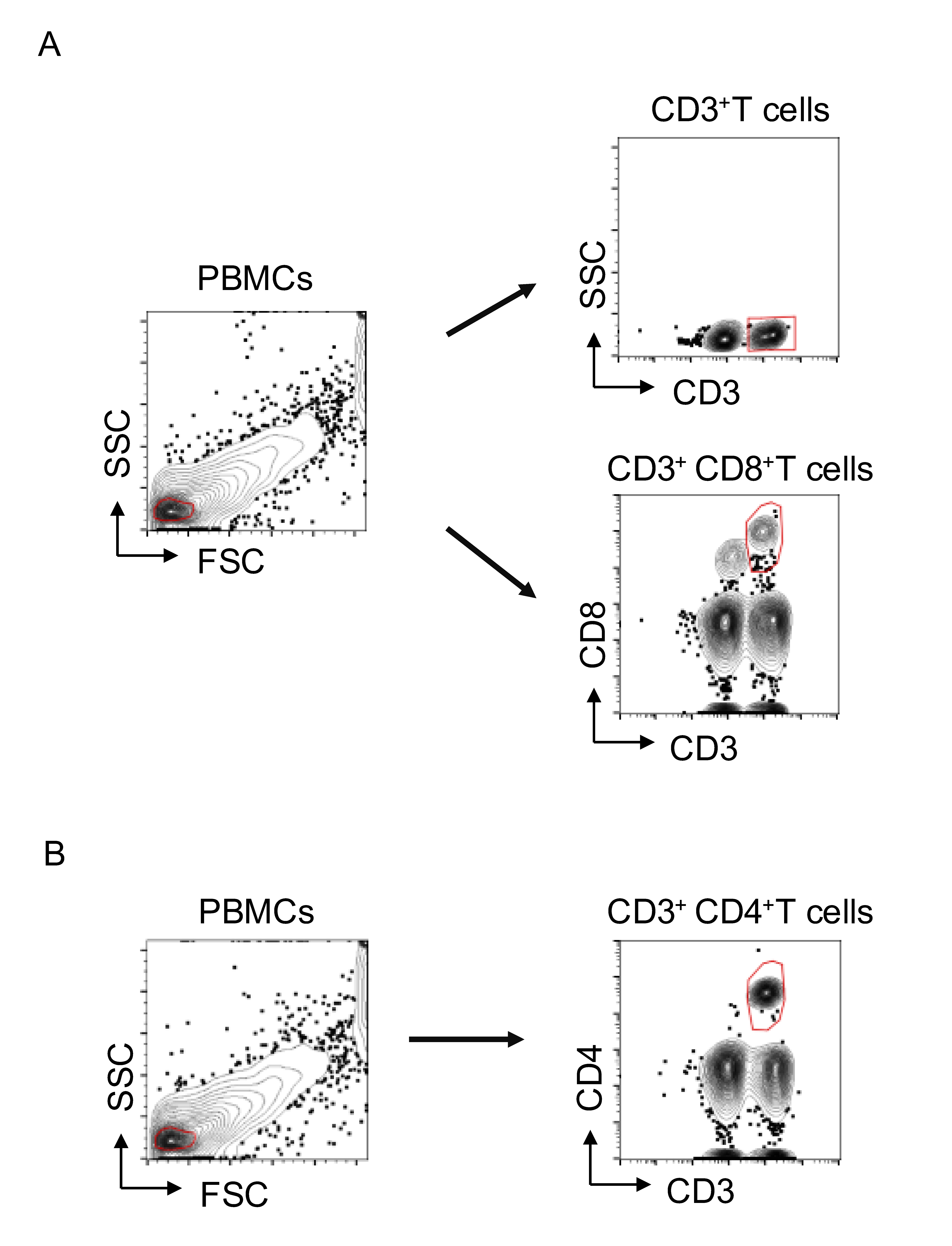

Supplement: Fig. S3 — Representative gating strategy for T-cell analysis. [file jvi.02014-25-s0004.tif]

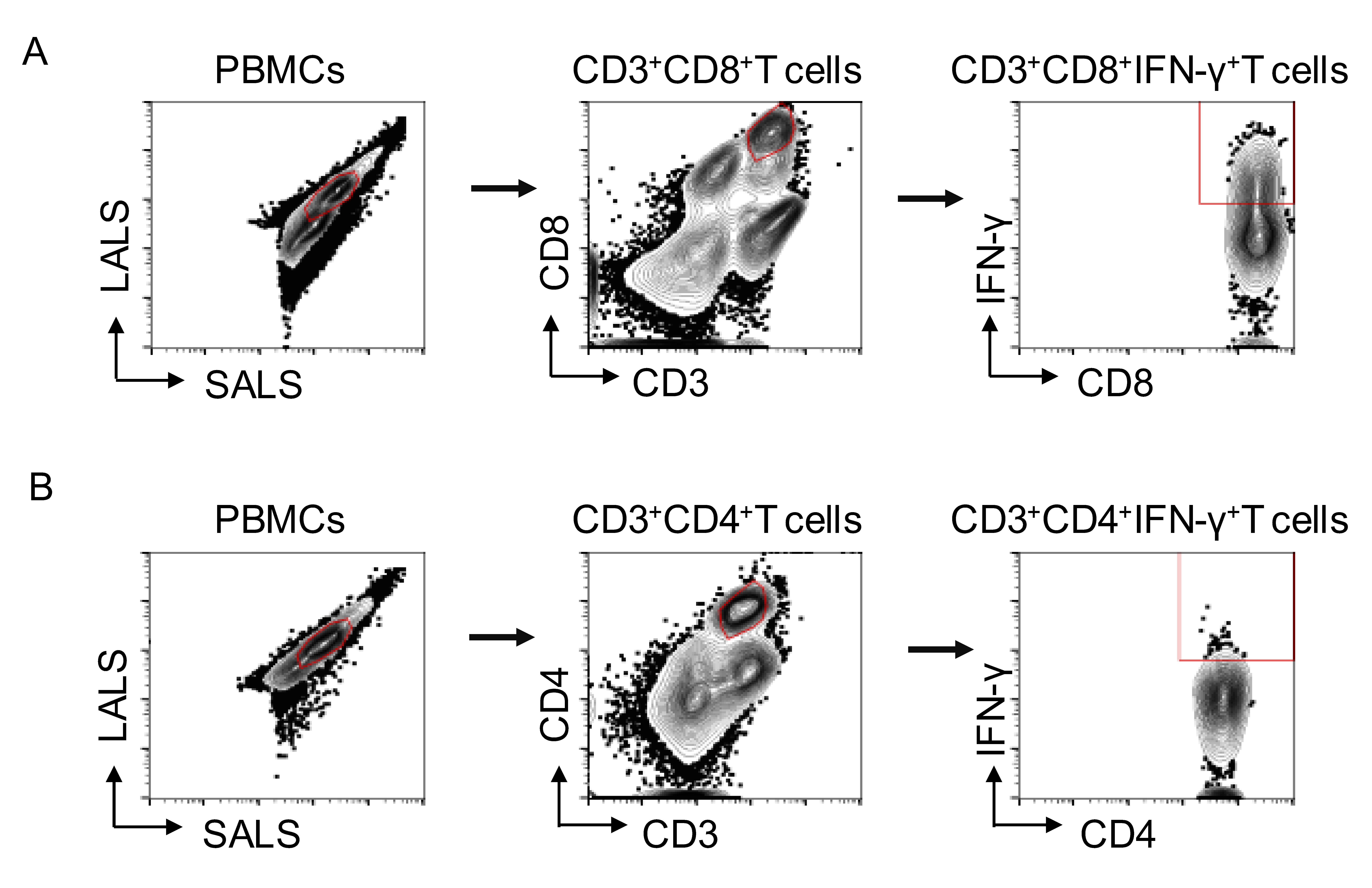

Supplement: Fig. S4 — Representative gating strategy for specific T-cell analysis. [file jvi.02014-25-s0005.tif]
