# Supplementary material for: Recombinant duck enteritis virus harboring the hemagglutinin genes of influenza virus rapidly induces specific cellular immunity in ducks
Source: J Virol. 2025 Dec 30;100(2):e02014-25. doi: 10.1128/jvi.02014-25 (PMC12911906; doi:10.1128/jvi.02014-25)
Supplement: Supplemental legends — Legends for Fig. S1 to S4. [file jvi.02014-25-s0006.docx]

**FIG. S1. Cytokine expression induced by rDEV-dH5/H7 and vDEV.**

The mRNA levels of (A) IFN-α, (B) IFN-β, (C) IFN-γ, (D) granzyme A, (E) IL-1β, (F) IL-2, (G) IL-4, (H) IL-6, (I) IL-8, and (J) IL-15 in PBMCs, bursa of Fabricius (BF), spleen and lung.

**Figure S2. Cross-reactivity and specificity of rat anti-CD3 mAb [CD3-12] with duck CD3.**

Duck or mouse CD3ε cDNA with a C-terminal Flag-tag was inserted into pCAGGS, yielding pCA-CD3ε _Duck_ and pCA-CD3ε _Mouse_; pCA-Flag (Flag only) served as negative control. HEK-293T cells transfected with the indicated plasmids were analysed by Western blotting (A) and flow cytometry (B). The rat anti-CD3 mAb [CD3-12] reacted with both duck and mouse CD3ε, whereas pCA-Flag and isotype IgG1 (Abcam, ab18404) controls remained negative. Flow-cytometric analysis of duck PBMCs, chicken PBMCs and mouse splenocytes confirmed that rat anti-CD3 mAb [CD3-12] binds CD3-positive lymphocytes from all three species(C).

Primer sequences used for amplification of duck and mouse CD3ε are as follows:

Duck CD3-F: 5’- ATC ATT TTG GCA AAG AAT TCG CCA CCA TGA GGT TTG AGC TGT CCT T -3’, Duck CD3-R: 5’- CCG TCA TGG TCT TTG TAG TCG TAG CCC CTG GAT TCC AGG C -3’.

Mouse CD3-F: 5’- ATC ATT TTG GCA AAG AAT TCG CCA CCA TGC GGT GGA ACA CTT TCT G -3’; Mouse CD3-R: 5’- CCG TCA TGG TCT TTG TAG TCG ACT GCT CTC TGA TTC AGG C -3’.

**FIG. S3. Representative gating strategy for T-cell analysis.**

Gating strategy for quantifying T cell subsets. PBMCs were stained as described and analyzed by flow cytometry. For each run, identical gates were applied to all the samples; for the present study, all the fluorescence gates were identical. Cells were progressively gated to identify (A) CD3^+^, CD3^+^CD8^+^ and (B) CD3^+^CD4^+^ T cells subsets, as shown in the rows.

**FIG. S4. Representative gating strategy for specific T-cell analysis.**

Gating strategy for quantifying antigen-specific T cells. Stimulated cells were stained as described and analyzed by flow cytometry. For each run, identical gates were applied to all the samples; for the present study, all the fluorescence gates were identical. Cells were progressively gated to identify (A) CD3^+^CD8^+^ and (B) CD3^+^CD4^+^ T cells subsets, as shown in the rows. Within those cells, individual gates for each IFN-γ were used.
